# Unveiling the 3D Spin Texture of Nanowires Using Integrated Microscopy Techniques

**DOI:** 10.1021/acs.nanolett.5c02395

**Published:** 2025-06-23

**Authors:** Claudia Fernández-González, Laura Álvaro-Gómez, Laura Fernández-García, Pamela Morales-Fernández, Lucía Gómez-Cruz, Muhammad Waqas Khaliq, Claire Donnelly, Michael Foerster, Miguel Ángel Niño, Eva Pereiro, Eduardo Martínez, Miriam Jaafar, Lucía Aballe, Lucas Pérez, Sandra Ruiz-Gómez

**Affiliations:** † ALBA Synchrotron Light Facility, Carrer de la Llum 2-26, Cerdanyola del Vallès, Barcelona 08290, Spain; ‡ Dept. Física de Materiales, 16734 Universidad Complutense de Madrid, Plaza de las Ciencias 1, Madrid 28040, Spain; ¶ Max Planck Institute for Chemical Physics of Solids, Nöthnitzer Str. 40, Dresden 01187, Germany; § 202533 Instituto Madrileño de Estudios Avanzados - IMDEA Nanociencia, C/Faraday 9, Madrid 28049, Spain; ∥ International Institute for Sustainability with Knotted Chiral Meta Matter (WPI-SKCM2), Hiroshima University, Hiroshima 739-8526, Japan; ⊥ Dpto. Física Aplicada. 16779 Universidad de Salamanca, Plaza de los Caídos s/n E-38008, 37008 Salamanca, Spain; # 69570 Instituto de Ciencia de Materiales de Madrid, CSIC, Cantoblanco, 28049 Madrid, Spain

**Keywords:** cylindrical nanowires, integrated microscopy, X-ray imaging, magnetic microscopy

## Abstract

This study integrates multiple microscopy techniques to investigate the effect of introducing axial compositional gradients on the magnetic properties of Fe–Ni ferromagnetic nanowires. We study the chemical structure of the nanowires using photoemission electron microscopy and transmission (X-ray and electron) microscopy. We explore the magnetic properties through magnetic force microscopy, complemented by micromagnetic simulations, and we characterize the 3D magnetization vector by measuring magnetic circular dichroism using X-ray microscopies. We observe that variations in the Fe/Ni ratio induce localized magnetic curling in Fe-rich regions, while Ni-rich segments predominantly exhibit axial magnetization, demonstrating how compositional gradients can control magnetic domain configurations on the nanoscale. Our results highlight the value of multimodal imaging for uncovering the interplay among structural, chemical, and magnetic properties in complex nanostructures. These findings represent a significant step toward the manipulation of magnetic domains in nanowires, which is essential for future devices based on 3D nanomagnetic architectures.

## Graphical abstract

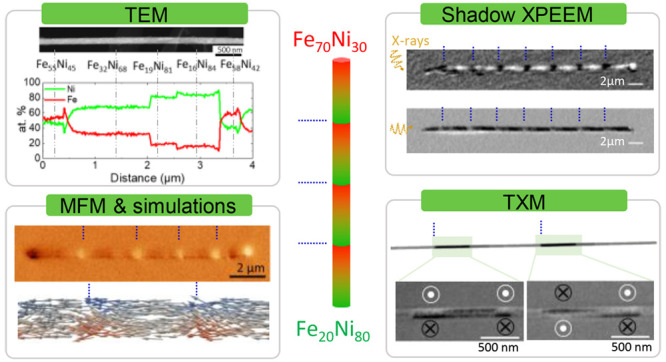

The progressive digitization of the world has led to the generation of a vast amount of data that must be stored, analyzed, and used efficiently. Given the physical constraints of superparamagnetism in magnetic recording, which limit the areal density of the current technology, the need for innovative approaches is increasingly evident. One of the most promising approaches to overcome these challenges is three-dimensional nanomagnetism, driven by advances in nanofabrication techniques that have enabled the fabrication of magnetic nanoarchitectures with unprecedented structural and functional complexity.^1^ These structures can accommodate complex spin textures such as vortex states, Bloch point domain walls, and magnetic skyrmions, which are stabilized or manipulated by curvature, confinement, and topology in 3D geometries.^2^ Such spin textures, which can be used as information tokens in logic and memory devices, exhibit emergent properties like topological protection,^3,4^ ensuring stability; fast dynamics,^5^ key for data transfer efficiency; and low depinning currents, essential for decreasing the energy consumption. These properties, combined with the inherent increase in data storage linked to the third dimension, offer a promising pathway toward energy-efficient, high-density memory, and logic technologies. To fundamentally understand the magnetic behavior and spin textures of complex 3D nanostructures, the integration of advanced imaging techniques is essential for resolving their structural, chemical, and magnetic configurations with high spatial resolution. Inspired by the success of correlative microscopy in life sciences,^6,7^ we propose the integration of multiple imaging techniques to bridge the gap between structural and functional information, leading to a better understanding of three-dimensional complex systems.

Transmission electron microscopy (TEM) offers high spatial resolution (up to 0.5 Å), enabling atomic-level observation and the precise identification of magnetic domains and defects.^8–10^ It also provides detailed chemical composition and crystal structure information through complementary techniques like energy-dispersive X-ray spectroscopy (EDS)^11^ and electron diffraction,^12^ essential for correlating magnetic properties with microstructure. However, TEM has significant disadvantages: sample preparation can be complex and destructive, potentially altering the original properties of materials, and techniques to obtain vector magnetization are relatively complex and time-consuming.

X-ray based microscopies such as X-ray photoemission electron microscopy (X-PEEM),^13,14^ transmission X-ray microscopy (TXM),^15,16^ ptychography,^17,18^ and holography,^19,20^ allow for high-resolution imaging—20–30 nm for X-PEEM and TXM, and down to ∼10 nm for ptychography and holography—and provide element-specific magnetic contrast due to the X-ray magnetic circular dichroism effect (XMCD), enabling detailed analysis of magnetic properties in multicomponent systems. These techniques enable nondestructive 3D imaging of magnetic domains and spin textures in thicker specimens than TEM. Furthermore, the pulsed nature of synchrotron radiation can be potentially used for time-resolved experiments. However, X-ray techniques require access to synchrotron radiation facilities.

Magnetic force microscopy (MFM)^21–23^ provides high-resolution magnetic imaging by detecting the magnetic forces between a sharp tip and the sample surface. This enables detailed mapping of magnetic domains and spin textures. Although they are excellent for high-resolution imaging which can reach down to 10 nm with specialized tips,^24^ and excellent sensitivity (approximately 10 pN), MFM also has a number of limitations that must be considered. One of the most significant is the lack of quantitative information, as accurately knowing the characteristics of the probe (e.g., its stray field distribution) is often difficult. Image interpretation is not trivial either, as it is a product of the convolution of the magnetic properties of both the tip and the sample. Additionally, other sources of artifacts exist, particularly electrostatic interactions between the tip and the sample. Finally, as a surface technique, obtaining in-depth information is not straightforward.

In this work, we propose the integration of these different microscopy techniques to study magnetic nanowires (NWs), a textbook case of a 3D nanomagnetic system, displaying all features of 3D but remaining simple and therefore easy to understand and model. We focus the study on Fe–Ni NWs with axial compositional gradients that enable tuning the domain wall (DW) energy landscape, leading to asymmetric magnetization processes.^25^ Beyond the inherent interest in tailoring the energy landscape for controlling domain wall dynamics, the unidirectional ratchet-like propagation of domain walls may underlie the design of shift registers^26^ and novel domain-wall-based logic devices.^27^ This article integrates multiple microscopy techniques to address the limitations of individual methods, correlating morphology, composition, crystal structure, and 3D magnetization in these ferromagnetic NWs with compositional gradients. Understanding the dependence of spin textures on the composition and structure is key for understanding the underlying physics and for their integration in future spintronics devices.

The NWs under study are 110 nm in diameter and 30 μm in length. They consist of a periodic repetition of Fe–Ni gradients, each 2 μm long, along the longitudinal axis of the wire, where the Ni/Fe ratio transitions from Ni/Fe < 1 to Ni/Fe > 1 (see Figure 1a). To access the chemical structure of the NWs, both spatial and chemical resolutions are required.

**Figure 1:**
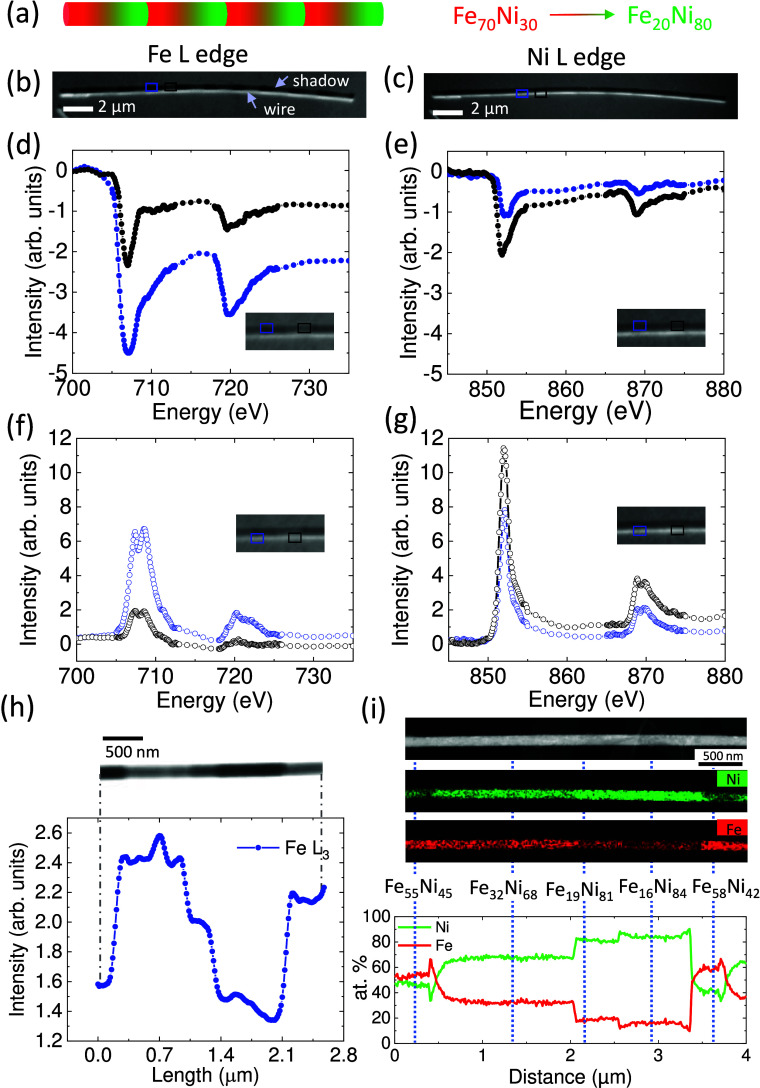
(a) Schematics of a NW with four chemical gradients, where composition ranges from Fe_70_Ni_30_ (represented in red) to Fe_20_Ni_80_ (represented in green). (b, c) X-ray absorption PEEM images taken at the Fe L_3_ and Ni L_3_ absorption edges of a ratchet-type Fe–Ni NW with a diameter of 110 nm. Normalized X-ray transmission (d, e) and absorption spectra (f, g) of the Fe L edge (left) and Ni L edge (right) extracted from the shadow region of the image (filled points) and the wire region of the image (unfilled points), respectively. Black plots are the spectra extracted from the Ni-rich region of the gradient and blue plots correspond to the Fe-rich region. (h) Top: X-ray transmission image taken at the Fe L_3_ edge on one of the chemical gradients of the NW using TXM. Bottom: plot of the transmitted intensity profile along the NW axis of the image. (i) HAADF STEM image and corresponding STEM-EDS compositional Fe (red) and Ni (green) maps of the NW. The plot represents the atomic percentage quantification along the axial direction of the NW.

Figure 1 shows a comparison of the chemical information obtained using different spectromicroscopy techniques, both X-ray and electron-based. The information from Figure 1b to 1g is obtained using shadow-PEEM. A XAS spectra showing the Fe and Ni L_3_ and L_2_ absorption edges can be extracted from images acquired at different photon energies. Information from the wire arises from electrons emitted from the surface of the NW (about 5 nm in depth), providing information from the surface of the NW whereas the information from the shadow corresponds to electrons photoemitted from the substrate, which are excited by the X-ray beam transmitted through the wire and, therefore, providing information from the volume of the NW. Thus, in a single measurement, bulk and surface information can be obtained and compared, albeit at a much lower resolution than in TEM. Figure 1b and 1c shows the X-ray absorption images taken at the Fe L_3_ and Ni L_3_ edge. For the Fe L_3_ edge, periodic contrast variations are strongly visible in the shadow region. Darker regions correspond to areas with higher photon absorption, indicating higher Fe concentration (blue square), while less dark areas correspond to regions with a lower Fe concentration (black square). In the case of the Ni L_3_ edge image, the chemical variation is more obvious on the wire surface. Here, brighter regions correspond to Ni-rich areas (black square), while less bright regions correspond to Ni-poor areas (blue square).

Full Fe and Ni L edge spectra can be extracted from image stacks acquired while the photon energy. Transmission spectra—extracted from the shadow—are shown in Figure 1d and 1e, and absorption spectra—extracted from the wire—are in Figure 1f and 1g. Spectra from Fe-rich regions are plotted in blue, and those from Ni-rich regions are plotted in black. Chemical composition can, in principle, be calculated from the intensity difference above and below the absorption edge, since this jump depends linearly on the number of absorbing atoms.^28^ However, the quality of the data in this case is limited by the small regions from which the spectra are obtained and by the angularly inhomogeneous photoemission from nonflat surfaces (such as NWs and their vicinity), leading to an experimental error of approximately 10%. From the Fe transmission spectra, we estimate a variation of the Fe content by a factor of 2.7× between both ends of the ratchet, whereas from the Ni absorption spectra, a factor of 2× in Ni content is obtained, values which indicate a gradient from about Fe_61_Ni_39_ to about Fe_23_Ni_77_, values not precise enough to ascertain e.g. variations of composition between surface and bulk. This limitation restricts the use of XPEEM for the quantification of elemental ratios in nanostructures where small signals are obtained. However, X-ray absorption spectra also give information on the oxidation state of the probed element. Both the Fe and Ni spectra on the wire surface show signs of oxidation (distinctly resolved double peak in Fe L_3_ of Figure 1f and in Ni L_2_ of Figure 1g), while the transmission (bulk) spectra do not.

Figure 1h shows TXM data, a photon-in-photon-out technique unaffected by sample curvature, in contrast to XPEEM. Here, the compositional variations are better resolved, as can be seen in the transmission image. As Fe content increases, absorption rises and transmitted intensity decreases from bright (low Fe) to dark (high Fe) regions. Contrast differences reflect segments with different composition.^25^ The plot below the image corresponds to the intensity profile along the wire and shows a ratchet pattern. Using the absorption edge jump extracted from Fe L TXM spectromicroscopy stacks, we also obtain a variation of the Fe content by a factor of 2.7 between both ends of the gradient with an associated error of 6%.

While XPEEM and TXM enable correlation of structural, chemical, and magnetic properties, TEM provides unsurpassed spatial resolution—down to the atomic level—essential for accurately resolving the chemical gradients. Figure 1i shows the HAADF image and EDS-STEM measurements. The compositional maps confirm the previous results obtained with XPEEM and TXM. The quantified atomic percentages in the lower part of Figure 1i reveal that Ni dominates along the gradient, except on both ends where Fe surpasses Ni. Vertical dotted lines correlate the compositional regions in the plot and maps with wire morphology in the HAADF image. In addition, TEM offers high-resolution details of the crystal structure. In this case, despite the changes of the Fe/Ni ratio along the NWs, they maintain a consistent polycrystalline fcc structure throughout the full compositional gradient.

To characterize the spin textures and magnetic behavior, various techniques have been employed, from MFM to X-ray methods. MFM measurements were performed to study how axial compositional gradients affect the magnetic properties. Figure 2a shows topography and phase contrast (magnetic) images of a NW. Periodical bright regions can be observed in the magnetic contrast image, matching the gradient length (Figure 2b). These changes in contrast correspond to different orientations of the stray field vector compared to the rest of the wire (areas with almost no contrast). Simulations were done to correlate the magnetic contrast with the chemical structure of the wire. Figure 2c shows the simulated amplitude (top) and phase contrast (bottom). The bright contrast in the phase image arises from the regions of the wire where the changes in the Fe/Ni ratio are stronger (yellow arrows in the amplitude image). This suggests that the direction of the stray field at these points differs from the rest of the wire. Figure 2e shows the 3D magnetization orientation predicted by micromagnetic simulations of the NW in remanence. The magnetic moments exhibit local deviations from the expected axial magnetization (i.e., for a homogeneous cylindrical wire) at the Fe-rich ends of the chemical gradients (yellow arrows). In these regions, due to the difference in *M*_s_, volumetric charges are generated to reduce the dipolar energy of the system, leading to the curling of the magnetization, which comes at the expense of exchange energy. As a result, magnetization tilts toward the azimuthal direction, forming short helicoidal segments at the regions where composition changes from Fe-rich to Ni-rich.^29^

**Figure 2:**
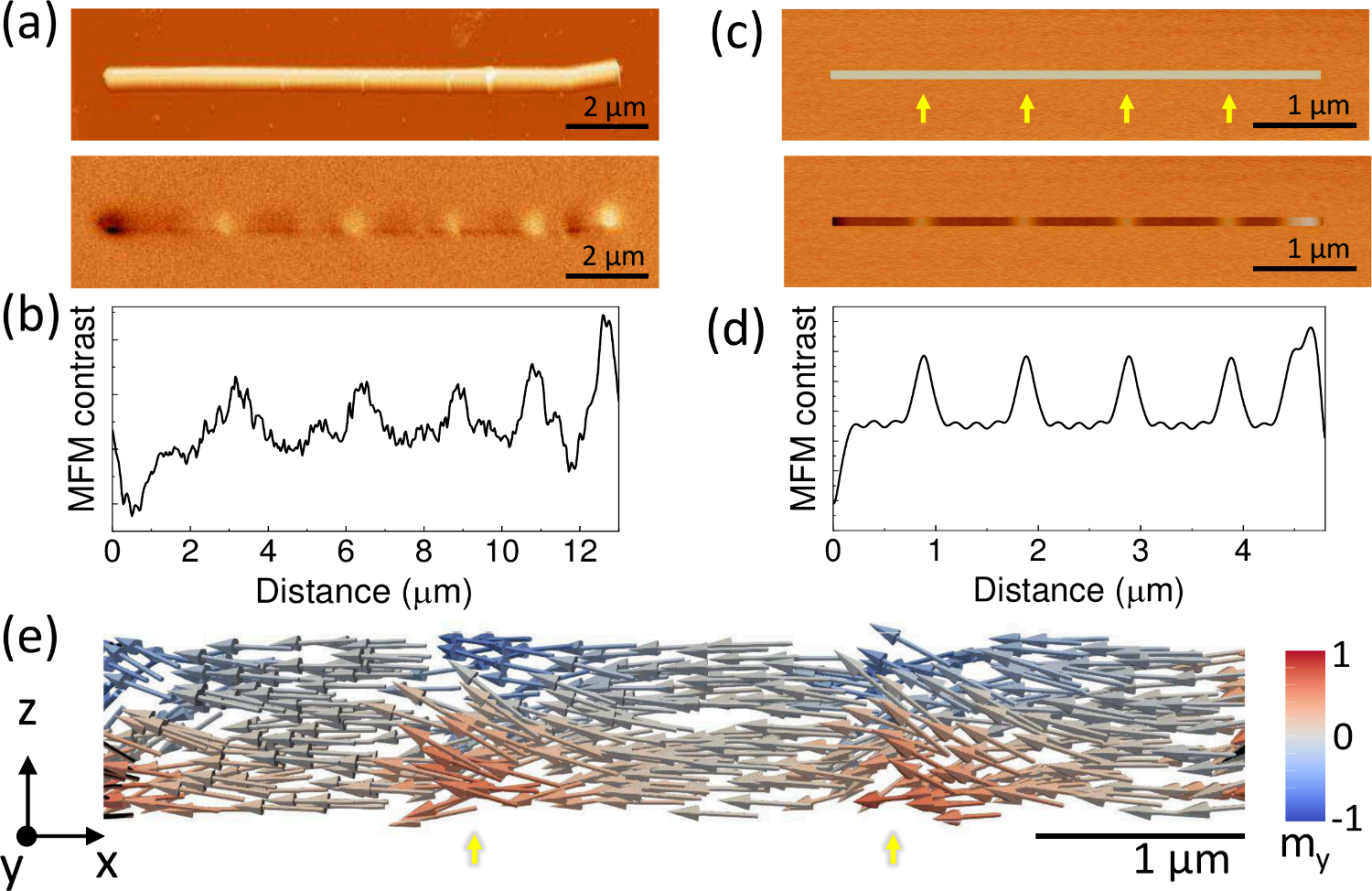
(a) AFM (top) and MFM (bottom) images of a NW with axial composition gradients of 2 μm in length. (b) Variation of the MFM contrast along the NW long axis. (c) Micromagnetic simulations of the AFM contrast (top) and MFM contrast (bottom) of a NW with axial gradients of 1 μm. (d) Variation of the simulated MFM contrast along the NW long axis. (e) Orientation of the magnetization vector predicted by micromagnetic simulations.

XMCD-PEEM was performed to verify the 3D magnetic configuration observed by MFM and predicted by micromagnetic simulations. Figure 3 shows the 3D magnetic characterization of the magnetization vector. As XMCD is only sensitive to magnetization components aligned with the X-ray direction (k⃗) , one can access the different components of the magnetization vector by rotating the sample and acquiring different projections (m⃗) , enabling its reconstruction.^13^ At 90° rotation (b), only components perpendicular to the NW axis are detected ( m⃗·k⃗ , where *k_x_*, *k_y_* ≠ 0, *k_z_* = 0), while at 45° and 5° (c, d), the axial component is detectable ( m⃗·k⃗ , where *k_x_*, *k_y_*, *k_z_* ≠ 0). The corresponding XMCD images are shown on the right side of the schematics (f–h). The yellow arrows highlight the regions where the Fe content drops from Fe_55_Ni_45_ to Fe_16_Ni_84_. In (f), the photon incidence is perpendicular to the right side of the wire, with the left side at about 85° due to a slight bend. The right side of the NW shows magnetic contrast in the regions corresponding to Fe/Ni > 1, where dark and bright contrast can be observed, indicating a radial component of the magnetic moments in these small regions. This magnetic curling induces volumetric charges of opposite sign to the surface charges at the material interfaces, reducing the total magnetic energy of the system, as mentioned above.^29^ Periodic contrast changes are also visible on the left side, along with magnetic contrast in the shadow and wire regions. This suggests a magnetization component along the wire axis, which shows no contrast at 90° incidence, and a perpendicular component influenced by the Fe content. When the sample is rotated more parallel to the incident photons (g), the axial component contributes more to the XMCD image. Magnetic contrast is observed on the right side of the wire, displaying values opposite those on the left side. This effect is particularly pronounced in (h), where the photon incidence is almost parallel to the axis of the NW. The opposing contrast between the left and right sections of the NW indicates the presence of opposite axial magnetic domains separated by a DW (green arrow).

**Figure 3:**
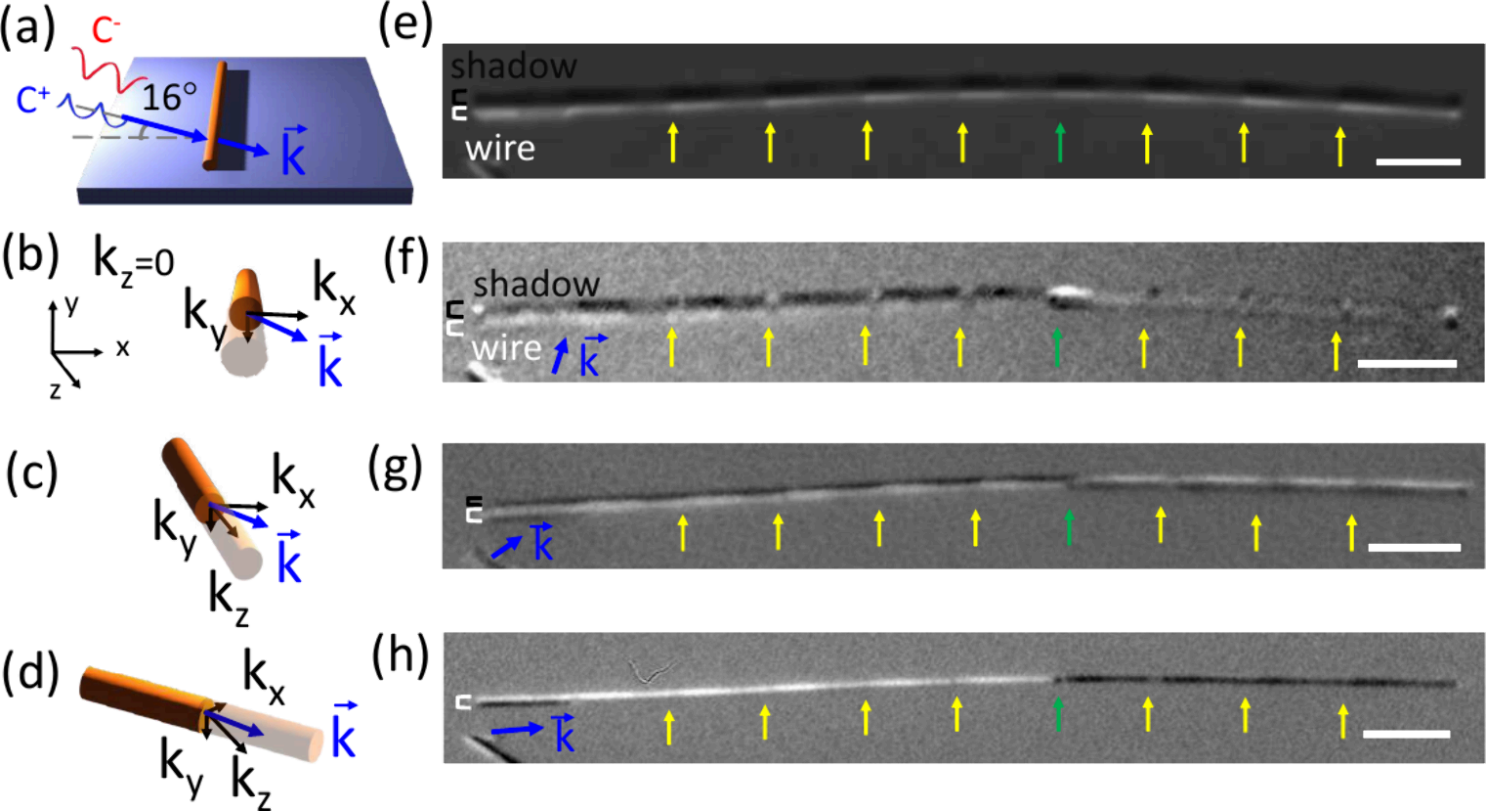
Magnetic characterization using shadow XMCD-PEEM. Schematics of the sample orientation respect to the incident photon beam for rotation angles of (a, b) 90°, (c) 45°, and (d) 5°. The wave vector *k⃗* points the direction of the incident photons, and *k*_*x*_, *k*_*y*_, and *k*_*z*_ are the unit components of *k⃗* in the coordinate system of the wire. (e) X-ray absorption image at the Fe L_3_ edge. (f–h) Corresponding magnetic contrast images for each of the angles. Scale bar is 2 μm in all images.

The resolution of the magnetic contrast image obtained with XPEEM is lower than that provided by TXM for very thin 3D samples like NWs. This limitation originates from edge-related artifacts in the XPEEM images, which can distort the magnetic signal near the NW boundaries. In contrast, these artifacts are absent in TXM images, as the transmitted X-ray photons pass through the sample and reach the detector without suffering significant distortion, allowing higher spatial resolution and a more accurate determination of the magnetization orientation.^30^

To study in more detail the orientation of the magnetization inside each segment, we took XMCD-TXM images in NWs with longer gradients (4.5 μm) at two different angles. Figure 4.b shows a wire segment with two gradients: yellow arrows indicate the ends of the gradients while blue arrows indicate the end of the section where Fe/Ni > 1. At 0° (Figure 4c), magnetic contrast appears between yellow and blue arrows and at the wire end (right part of the image), arising from the magnetization components perpendicular to the wire. In these Fe-rich regions contrast changes in the transversal direction, from dark on the bottom to white on the top. This means that the magnetization is circulating around the NW axis; this circulation is also observed at ends of the wire, corresponding to a closure domain. If we zoom in the Fe-rich segments (Figure 4e and 4f), some differences can be observed. In the left Fe-rich region (e), the direction of the circulation of the magnetization is the same on the edges of the segment, while the central part shows a domain with an axial component of the magnetization opposite that observed in the Ni-rich regions. In the Fe-rich region on the right (f), two azimuthal domains with opposite circulation can be observed, separated by a DW. The rest of the wire shows weak contrast at 0°, indicating that the component of the magnetization perpendicular to the wire axis is almost nonexistent. In the image taken at 15° (Figure 4d), the axial component of the magnetization is readily detectable as white contrast in Ni-rich regions, which means that the magnetization points in the same axial direction along the full visible wire. Magnetization is therefore mainly axial, but when Fe/Ni > 1, it curls generating azimuthal magnetic domains due to a local increase in the *M*_*s*_ confirming the results obtained with micromagnetic simulations.

**Figure 4:**
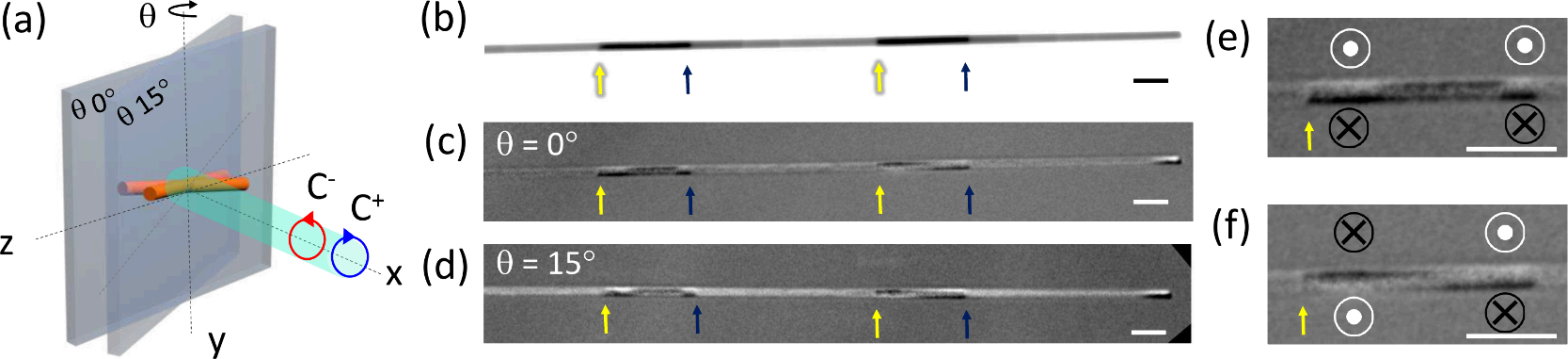
(a) Schematics of sample and X-ray orientation inside the TXM microscope. θ indicates the rotation angle. (b) Transmission image taken at the Fe L_3_ edge. (c, d) XMCD images taken at the Fe L_3_ edge with the sample at 0° and rotated at 15°, respectively. (e) Zoom of the area with curling in the magnetization on the left side of image (c). (f) Zoom of the area with curling in the magnetization on the right side of image (d). Scale bar is 500 nm in all images.

We have therefore explored the interplay between structure, composition, and magnetization in ferromagnetic NWs with axial gradients by integrating X-ray, electron, and scanning-probe microscopies to successfully correlate their morphology, composition, crystal structure, and 3D magnetic configuration.

Specifically, with XPEEM and TXM we were able to characterize the magnetization vector of the NWs, which shows magnetic curling in the regions of high Fe content. XPEEM provided insights into both surface and bulk chemical compositions, and TEM delivered atomic-scale structural information. The combination of TEM (for morphology) and XPEEM (for surface and chemical mapping) allowed us to determine that the observed changes in magnetization arise from compositional gradients rather than from changes in crystallinity or in the effective thickness, coming, for example, from different oxidations in Fe-rich or Ni-rich areas. While surface shell oxidation can, in principle, be distinguished using TEM, this becomes significantly more challenging in samples with thicknesses above 100 nm. MFM allows us to measure the stray field emerging from the NWs in a relatively simple manner (no need for vacuum or an X-ray facility) and with high resolution but requires micromagnetic simulations to understand the observed contrast. XMCD-PEEM and TXM allow for direct imaging of the projection of magnetization, simplifying the analysis and providing element-resolved magnetization. In addition, full characterization of the magnetization vector is possible in both XPEEM and TXM by acquiring images at different angles, ascribing the magnetization curling to the higher Fe content areas.

One of the main limitations of integrating multiple microscopy techniques lies in the lack of a universal substrate compatible with all methods, which would facilitate the characterization of the exact same sample, i.e., a single NW or nanostructure. Designing such a substrate for correlative microscopy remains a significant challenge as it must meet the diverse requirements of various techniques. For instance, XPEEM necessitates a highly conductive substrate, whereas transmission-based microscopies require substrates that are both X-ray- and electron-transparent. Considerable efforts are currently being devoted to developing sample environments that can accommodate these often incompatible demands.

Overall, the ability to precisely control and analyze the magnetic properties of nanostructures through compositional engineering opens new avenues for future research and technology development. In this sense, new advances in integrated microscopy approaches, including correlative microscopy under operando conditions, as well as time-resolved experiments, will be essential for paving the way to new applications. In the particular case of the studied NWs, microscopy under current pulses would help to visualize the asymmetric domain wall motion, correlating the threshold currents and time with the compositional gradients, and time-resolved experiments would allow for the accurate measurement of domain wall velocities and explore the possibility of reaching the magnonic regime in these systems.

## Experimental Section

NWs were synthesized using template-assisted electrodeposition, as described in ref 25. To create the chemical gradients, the growth potential was changed from −0.9 V to −1.5 V (vs Ag/AgCl). After growth, NWs were released from the template by chemical etching in a solution of 0.4 M H_3_PO_4_ and 0.2 M H_2_CrO_4_ and kept in EtOH 99.5% vol.

TEM measurements were performed on a JEOL JEM 3000F microscope. NWs were drop-cast onto a holey carbon-coated grid. High-angle annular dark-field images (HAADF-TEM) were acquired and X-ray energy dispersive spectroscopy (EDS) measurements were performed on the Fe Kα and Ni Kα transitions to obtain the elemental mapping of the sample with atomic resolution.

For MFM, a Nanotec Electronica microscope controlled by WSxM software^31^ and a Bruker Nanoscope6 microscope, equipped with a magnetic field application stage, were used. The measurements were carried out in amplitude modulation mode, thus, recording the magnetic signal as the phase of the oscillation. The probe used was a Nanosensors PPP-MFMR. The scanning direction was set perpendicular to the NW axis with a 150 nm lift. NWs were drop-cast on top of Silicon substrates for MFM measurements.

For XPEEM, NWs were drop-cast on conductive substrates and demagnetized prior to the measurements. X-ray absorption spectroscopy (XAS-PEEM) was measured at the Fe L and Ni L edges using circular left (CL) and circular right (CR) polarized photons, and low-energy secondary electrons were collected to form images. XAS-PEEM image stacks with lateral resolution of tens of nanometers were acquired at the CIRCE beamline of the ALBA Synchrotron.^32^ (Full field) TXM measurements were performed at the MISTRAL beamline of the ALBA Synchrotron.^33^ In this beamline, the X-ray source is a bending magnet, so CR and CL polarized photon beams were obtained by modifying the electron orbital to have a descending or ascending trajectory in a short section of the storage ring. The microscope was operated with a magnification of ×1300 to provide an effective pixel size of 10 nm in the transmitted images. For TXM measurements, NWs were deposited on 50 nm thick Si_3_N_4_ membranes transparent to X-rays. XMCD images were measured at the Fe L_3_ absorption edge both in PEEM and in TXM. Magnetic contrast images were obtained by a pixel-by-pixel subtraction of images acquired with opposite photon helicity at the same resonant X-ray absorption energy.

Micromagnetic simulations were performed with the mumax3 code,^34^ in a 90 nm diameter wire with total length of 4.8 μm length and a 1 μm chemical gradient. Finite element discretization size was set to 3 nm. The exchange constant was *A*_ex_ = 13 × 10^–12^ J/m, damping constant α = 1, and saturation magnetization gradually changed from μ_0_ *M*_s_ 800 × 10^3^ A/m to 1400 × 10^3^A/m. MFM simulations were performed with a vertical magnetized tip and a scanning lift of 200 nm.
